# Epibulbar Osseous Choristoma

**DOI:** 10.1155/2014/292619

**Published:** 2014-12-15

**Authors:** Tolga Bicer, Hasan Soylemez

**Affiliations:** ^1^Ophthalmology Department, Diskapi Yildirim Beyazit Education and Research Hospital, Irfan Bastug Street, Diskapi, 06330 Ankara, Turkey; ^2^Radiology Department, Unye Cakirtepe Hospital, Atatürk Mah. Tepeyolu Street No. 43, 52300 Ordu, Turkey

## Abstract

The topic of this case report is a rare subconjuctival osseous choristoma that corresponded to the left lateral sunconjunctiva and canthus. A 20-year-old man was asymptomatic when he arrived for the examination. His full ophthalmic examination was normal. Orbital computerized tomography was concordant with osseous lesion. Osseous choristomas are the rarest forms of ocular choristomas, they are usually being defined as sporadic, and they are found at the superior temporal region of the episclera. In our case, choristoma was in the lateral canthus of the left eye. We had administered surgical excision by reason of the patient's cosmetic requirement. We had noted that the lesion was adherent to conjunctiva but not to the sclera and the muscles. After surgical treatment, we saw mature heterotrophic osseous tissue in subconjunctival area and Haversian canals in compact bone tissue.

## 1. Introduction

Osseous choristomas are solid nodules, composed of mature, compact bone together with pilosebaceous units and hair follicles. The topic of this case report is a rare osseous choristoma that localized to the left lateral subconjunctiva, in which underlying sclera was not involved.

## 2. Case Report

A 20-year-old man was asymptomatic when he arrived for the examination. In his medical history, he had a swelling on the left corner of the left eye since his birth. He had no medical problems or trauma. He has never used alcohol; however, he was smoking. His family thought that there was growth in the lesion compared to the past. His visual acuity was 20/20. External examination of the right eye, anterior segment, and fundus examination of both eyes were normal.

**Figure 1:**
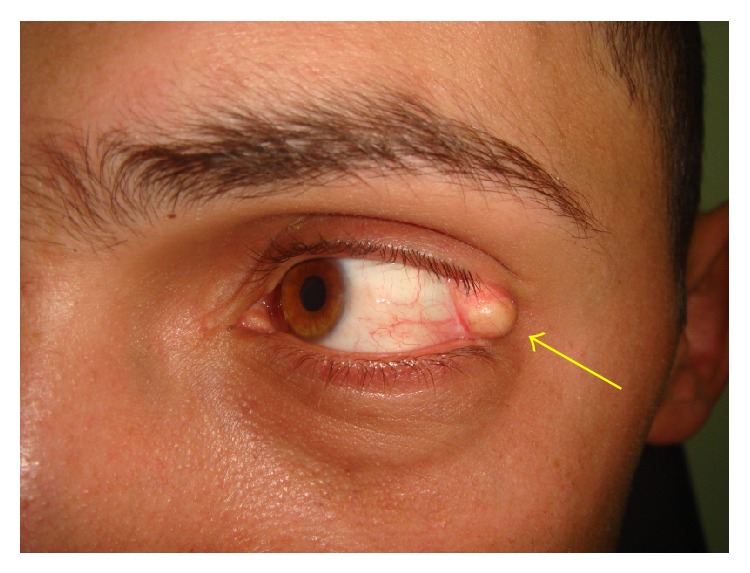
Subconjunctival lesion of the left eye (arrow).

**Figure 2:**
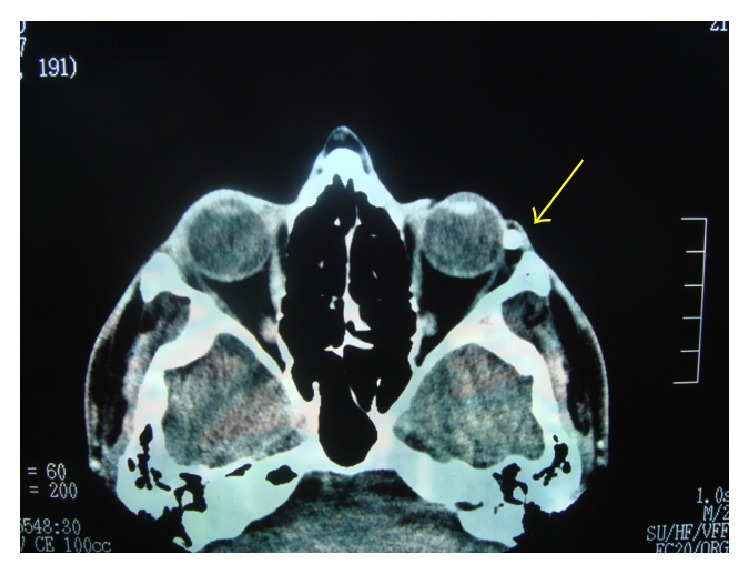
Episcleral osseous choristoma on the lateral canthus of left eye in computed tomography (arrow).

The swelling was approximately 6.0 mm × 6.0 mm subconjunctival lesion on the left eye (Figure 1). It was a mobile mass going towards left lateral canthus, so that we thought it did not involve the underlying sclera. The swelling was firm and had hair follicles and vascularization on some areas. Orbital computerized tomography was concordant with osseous lesion (Figure 2).

**Figure 3:**
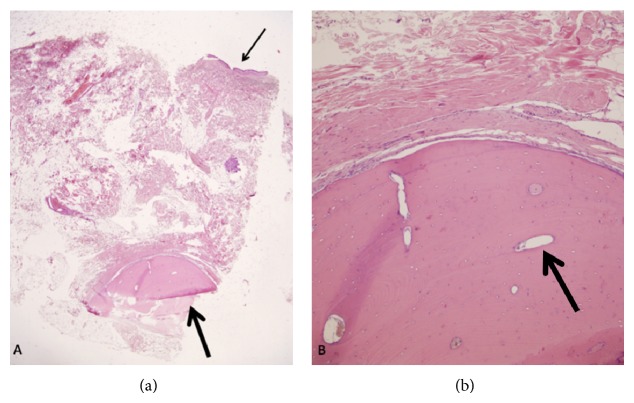
(a) Conjunctival epithelium (thin arrow) and heterotrophic osseous tissue (left thick arrow) (HEx20); (b) osseous tissue-like compact bone and Haversian canals (HEx200) (right thick arrow).

Informed consent was obtained for the local anesthesia. Intraoperatively, the lesion was adherent to conjunctiva but not to the sclera and the muscles. The mass and the adherent conjunctiva were removed totally and the remaining conjunctiva tissue was stitched edge to edge. The swelling was sent to histopathology immediately. Its result was episcleral osseous choristoma. Histopathologically, mature heterotrophic osseous tissue was seen in subconjunctival area (Figure 3(a)).

## 3. Discussion

The term heterotopia (choristoma) is applied to microscopically normal cells or tissues that are present in abnormal locations. Examples of heterotopias include the rest of pancreatic tissue found in the wall of the stomach or small intestine; a small mass of adrenal cells found in the kidney, lungs, ovaries or elsewhere; or as in our case a mass of mature bone cells found under conjunctiva. It was first described by von Graefe as epibulbar osteoma [1, 2]. Ocular choristomas consisted of limbal dermoids, dermolipomas, complex choristomas, and osseous choristomas [3, 4].

In a 302-periorbital-tumor-case series, Elsas and Green reported that 33% were choristomas, 29% were nevi, 11% were epidermal inclusion cysts, and 7% were papillomas in children. In the study, there were approximately 100 choristomas; 58% of the choristomas were dermoid cysts, 30% were dermolipomas, 6% were dermoid choristomas, and 6% were teratomas [5].

The other reported localizations of extraocular choristomas were the oral cavity, usually in the tongue and also at other soft tissues of the head and neck [6].

The differential diagnosis includes epibulbar dermoids, epithelial inclusion cysts, limbal dermoids, prolapsed orbital fat, papillomas, dermolipomas, and complex choristomas.

Osseous choristomas are the rarest forms of ocular choristomas, they are usually being defined as sporadic, and they are found at the superior temporal region of the episclera [4, 7]. Gayre et al. analyzed 51 cases of epibulbar osseous choristomas and reported that 69% were female, 76% were in the right eye, and 74% had superotemporal localization preponderance [8]. Our case was 20-year-old male whose choristoma was in the lateral subconjunctiva of his left eye. His gender and the affected eye were a different property when compared with general literature.

In literature, there are very few pieces of information about hair follicles localized in the conjunctiva, overlying the choristoma. In our case there were multiple hair follicles, localized only in the overlying conjunctiva of the choristoma. Intraoperatively, the mass and the adherent overlying conjunctiva were excised and the rest of the conjunctival tissue was stitched edge to edge.

The lesions are generally adherent to extraocular muscle sheath, overlying conjunctiva or underlying sclera [9]. We had noted that the lesion was adherent to overlying conjunctiva but not to the sclera and the muscles. It was a different property when compared with the general literature.

Surgical excision is needed when choristomas get symptomatic and also for cosmetic reasons, although choristomas are stable lesions and usually grow slowly [3, 4, 7, 8]. We had administered surgical excision for the reason of the patient's cosmetic requirement.

Histopathological images of the choristomas show mature compact bone, surrounded by fibrous connective tissue [4, 7]. Typically lesions contain Haversian canals. Although there are a few case reports describing the presence of hematopoietic tissue, no bone marrow tissue has been seen [8]. In our case, mature heterotrophic osseous tissue was seen in subconjunctival area (Figure 3(a)) and Haversian canals in compact bone tissue (Figure 3(b)) which was typical for this type of choristoma.

In our case, the choristoma was localized in the left eye, was not adherent to the underlying sclera and the muscles, and had hair follicles, contrary to general literature articles. As a result, rarely seen episcleral osseous choristoma should be considered when a mass is seen in any eye, gender, and age, especially localized superotemporally.
